# Longitudinal three-photon imaging for tracking amyloid plaques and vascular degeneration in a mouse model of Alzheimer’s disease

**DOI:** 10.1117/1.JBO.31.1.016004

**Published:** 2026-01-02

**Authors:** Eline Stas, Mengke Yang, Simon Schultz, Mary Ann Go

**Affiliations:** Imperial College London, Department of Bioengineering and Centre for Neurotechnology, London, United Kingdom

**Keywords:** three-photon microscopy, multicolour, longitudinal, vasculature, amyloid plaques, image segmentation

## Abstract

**Significance:**

Vascular abnormalities may contribute to amyloid-beta accumulation and neurotoxicity in Alzheimer’s disease (AD). Monitoring vascular degeneration as AD progresses is essential. Three-photon fluorescence microscopy enables high-resolution deep tissue imaging with minimal invasiveness and photodamage.

**Aim:**

In this proof-of-concept study, we established a longitudinal 3P imaging pipeline to quantify vascular and amyloid plaque changes in the ${\mathrm{APP}}^{\mathrm{NL}\text{-}G\text{-}F}$ mouse model.

**Approach:**

A cranial window allowed repeated 3P imaging at 4-week intervals beginning at 5 weeks after surgery. Vessels labeled with Texas-Red were segmented using DeepVess, whereas plaques labeled with methoxy-XO4 were segmented using custom scripts. Quantitative analyses assessed vascular parameters (diameter, tortuosity, length, inter-vessel distance, total volume) and plaque metrics (radius, total volume).

**Results:**

We imaged the same field over 4 weeks, quantifying an overall decrease in vasculature and an increase in amyloid plaques between two sessions. Significant changes in vessel diameter, inter-vessel distance, and alterations in vessel length and plaque radius were observed. Changes in vessel tortuosity were not significant.

**Conclusions:**

We demonstrate the potential of three-photon imaging to track vascular and amyloid-related changes in deep cortical structures. It offers a tool for studying the interplay between vascular and amyloid pathologies in AD, supporting future research into disease mechanisms and therapeutic strategies.

## 1 Introduction

Alzheimer’s disease (AD) is the leading cause of dementia worldwide, impacting the quality of human life and imposing significant healthcare costs.^1^ A widely accepted theory, the amyloid hypothesis, suggests that cognitive decline in AD patients is partly due to the accumulation of extracellular amyloid beta (Aβ) peptides in the brain.^2^ This accumulation is believed to trigger a cascade of pathological events, including the formation of intracellular neurofibrillary tangles composed of hyperphosphorylated tau and subsequent neuronal loss.^3^^,^^4^ Nonetheless, although amyloid pathologies have been the primary focus, emerging studies highlight the vascular system as a major factor in the progression of the disease.^5^

To meet its high energy demands, the brain relies on a constant supply of oxygen and energy substrates. As the brain has no long-term energy storage, regional cerebral blood flow is precisely regulated to align with the local energy requirements of nervous tissue. Any disruption in this adaptive process or in the delivery of necessary substrates can lead to imbalances in homeostasis, tissue damage, and functional impairment.^6^ These vascular abnormalities have been shown to not only contribute to Aβ accumulation, but also exacerbate neurotoxicity in AD.^7^^–^^9^ It is believed that the interplay between vascular degeneration and Aβ deposition is central to understanding the pathophysiology of AD progression. Brains of human AD patients commonly exhibit distinct vascular anomalies, including significant reductions in vascular diameter,^10^ density,^11^ and blood flow,^12^ whereas vessel tortuosity and length tend to increase.^13^^,^^14^ However, the precise mechanisms by which vascular abnormalities contribute to amyloid plaque buildup and subsequent neurotoxicity in AD remain unclear.^15^

Animal models are crucial for elucidating the pathophysiological pathways of AD and developing preventive, diagnostic, and therapeutic strategies. Although wild-type mice do not form senile plaques or neurofibrillary tangles,^16^ genetically engineered mice consisting of familial AD (FAD)-linked mutations in genes such as amyloid precursor protein (APP), presenilin1 (PSEN1), and PSEN2 are employed to mimic amyloidosis features of FAD-AD.^17^ Most AD cases are caused by sporadic AD; however, no animal models mimicking this form have been developed yet. Early transgenic models overexpressed APP, leading to excessive Aβ production and accumulation of APP fragments.^18^ However, APP overexpression can introduce artifacts not representative of human pathology.

The ${\mathrm{APP}}^{\mathrm{NL}\text{-}G\text{-}F}$ knock-in mouse model, which avoids APP overexpression by introducing specific mutations into the endogenous APP gene, provides a more accurate representation of preclinical AD.^19^^,^^20^ This model incorporates the Swedish (NL), Arctic (G), and Iberian (F) mutations, with homozygous mice exhibiting greater Aβ accumulation starting at 2 months than heterozygous ones starting at 4 months.^21^^,^^22^ It is considered a standard for studying mechanisms of Aβ amyloidosis.^18^ FAD typically manifests in humans in the heterozygous state; therefore, the heterozygous ${\mathrm{APP}}^{\mathrm{NL}\text{-}G\text{-}F}$ mutation more accurately models human pathology.^23^ Although other AD mouse models such as APP/PS1, 5xFAD, and APP23 have been utilized to study changes in vascular morphology and plaque progression,^24^^–^^27^ chronic imaging of vascular and amyloid interactions in the ${\mathrm{APP}}^{\mathrm{NL}\text{-}G\text{-}F}$ knock-in model has not been thoroughly investigated. This gap presents an opportunity to examine the relationship between vascular abnormalities and amyloid plaque formation in a model that more closely replicates human Aβ pathology.

Despite advancements in AD research, the disease’s complex pathogenesis remains poorly understood, highlighting the necessity for advanced longitudinal imaging methods to sequentially visualize brain structures and gain further insights into the mechanisms of the disease. Advanced imaging techniques are essential for studying the dynamics of vascular degeneration and amyloid pathology *in vivo*. Three-photon fluorescence microscopy (3PM) has significantly extended the penetration depth of high-resolution optical imaging, enabling the visualization of individual cells in preclinical rodent models with high spatial and temporal resolution.^28^ Compared with traditional noninvasive imaging techniques such as computed tomography (CT), magnetic resonance imaging (MRI), and positron emission tomography (PET), 3PM provides cellular-level resolution at the cost of requiring surgical cranial access and the use of high-power laser excitation. Nevertheless, when compared with other optical microscopy approaches, 3PM achieves deep-tissue imaging with relatively minimal photodamage.^29^^–^^32^ In addition, 3PM employs pulsed laser sources that deliver high peak power for excitation at low repetition rates, resulting in low phototoxicity.^33^

Specifically, 3PM enables deep tissue imaging using long excitation wavelengths (1300 to 1700 nm) and low average power, minimizing photodamage and phototoxicity.^33^^,^^34^ This approach reduces out-of-focus signal and enhances the signal-to-background ratio (SBR), allowing imaging at depths up to 1.4 mm and surpassing the limitations of two-photon fluorescence microscopy (2PM), which is often restricted to depths of 700  μm.^31^^,^^35^ Multicolor 3PM, using fluorescent dyes and genetically encoded markers, has successfully imaged structures such as GCaMP6s-labeled neurons, Texas-Red-labeled blood vessels, and third-harmonic generation (THG) signals from myelinated axons up to 1.1 mm deep. THG is a nonlinear optical phenomenon that enables label-free brain imaging by generating a signal at three times the original laser frequency, with its efficiency enhanced by structural discontinuities within the focal volume.^36^ However, simultaneous multicolour imaging of vasculature and Aβ using 3PM has not been demonstrated, and longitudinal imaging using 2PM has been limited to superficial cortical regions within layer five.^30^^,^^37^

Our study aims to address this gap by establishing a longitudinal imaging methodology for deep cortical structures in the brain, utilizing a 3PM imaging pipeline to investigate vascular and amyloid pathologies in the ${\mathrm{APP}}^{\mathrm{NL}\text{-}G\text{-}F}$ knock-in mouse model. By leveraging the deep imaging capabilities of 3PM, we seek to enable future studies to explore the dynamics of vascular degeneration and Aβ plaque accumulation. This approach will provide more accurate and translatable insights into the physiological mechanisms underlying AD progression, ultimately supporting the development of effective preventive and therapeutic strategies. Understanding these mechanisms could significantly impact future research and clinical practices in AD, paving the way for improved diagnostic and therapeutic approaches.

## 2 Methods

### 2.1 Three-Photon Microscope Setup

**Fig. 1:**
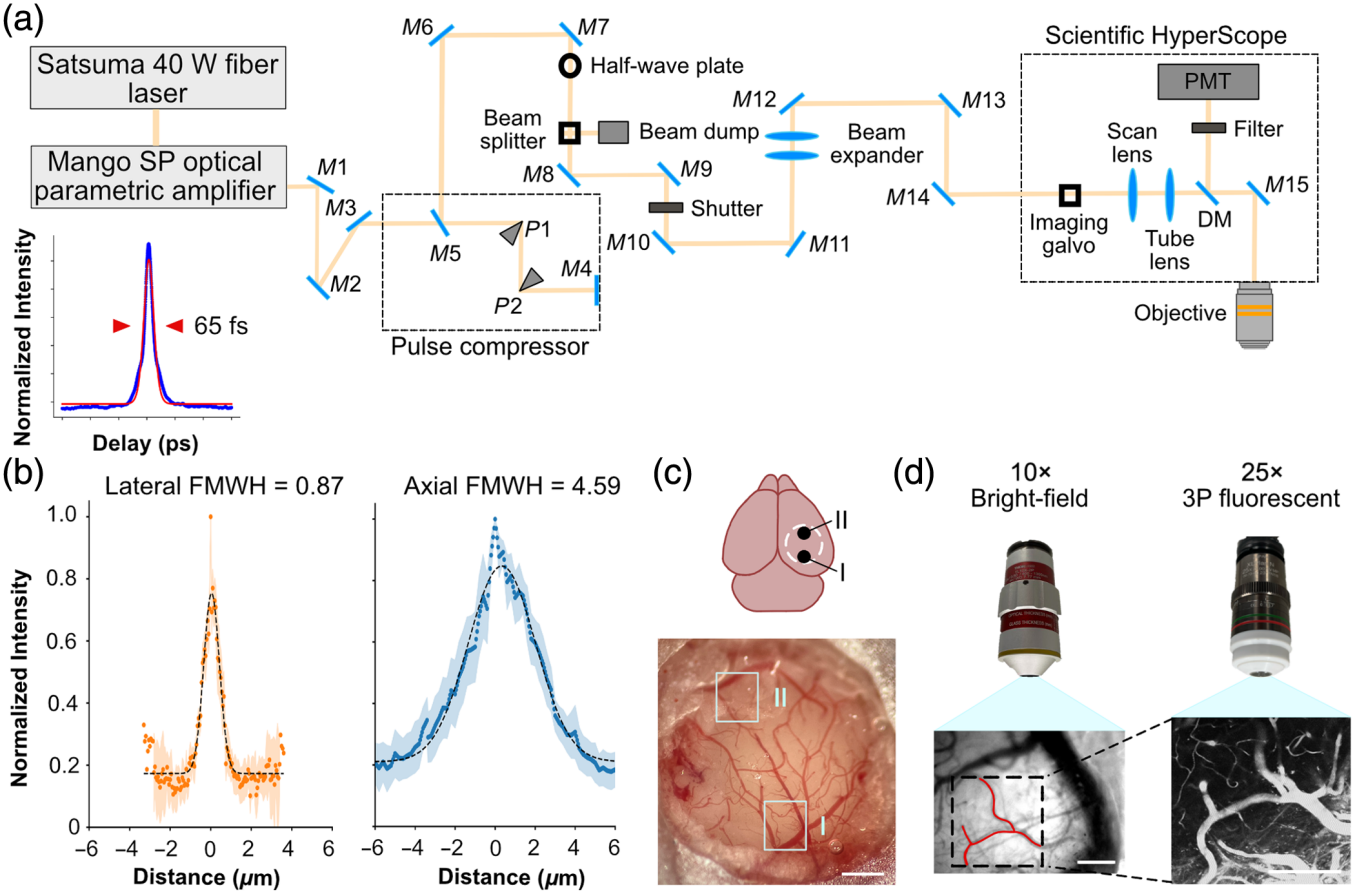
Imaging pipeline using a three-photon fluorescent microscope. (a) Schematic of the three-photon excitation imaging system with a pulse width of 65 fs. The system uses a Satsuma 40 W fiber laser and Mango SP optical parametric amplifier as the excitation sources. Key components include mirrors (M1 to M15), a prism pulse compressor (P1 to P2), a half-wave plate, a beam splitter, a beam expander, a shutter, a dichroic mirror (DM), scan and tube lenses, and an objective lens. The fluorescence signal is collected using a PMT. (Lower left) Measured pulse width shown in blue with ${\mathrm{sech}}^{2}$ fit (pulse width 65 fs) shown in red. (b) Intensity profile in the lateral (orange) and axial (blue) distance of 200-nm fluorescent beads (n=5). (c) Cranial window preparation and imaging locations: Diagram and bright-field image of a mouse brain with a cranial window. Imaging FOVs (I: ML: 2.25 mm, AP: −3  mm and II: ML: 2.25 mm, AP: −1  mm) are marked. Scale bar: 1 mm. (d) Imaging procedure and objectives: Bright-field and three-photon fluorescent images are acquired using 10× and 25× objectives to create vascular maps. Scale bars: 200  μm.

The 3P microscope [Fig. 1(a)] consisted of a 40 W fiber laser (Satsuma, Durham, North Carolina, United States), an optical parametric amplifier (OPA, Mango, Barcelona, Spain) running at a repetition rate of 1 MHz, and a hyperscope (Scientifica, Uckfield, East Sussex, United Kingdom). The laser pulses, with a width of 65 fs at 1340 nm, passed through a pulse compressor composed of mirrors (M3, M4, M5) and prisms (P1, P2). The beam was then directed through a series of mirrors (M1 to M15), a half-wave plate, and a beam-splitter for beam conditioning. The final power under the objective ranged between 165 and 220 mW. A tiltable objective mount was used, supporting a 25× water-immersion objective (XLPlan NA = 1.0, Olympus, Tokyo, Japan), with an ultrasound gel as the immersion liquid. The signals were divided into two detection channels using two photomultiplier tubes (PMTs) and dichroic beam splitters with specific wavelength filters (ET440/40 and ET620/60 from Thorlabs, Newton, New Jersey, United States). The ET440/40 filter was used to detect amyloid plaques via methoxy-XO4 fluorescence,^38^ whereas the ET620/60 filter was used to detect vessels via Texas-Red fluorescence.^36^ Before reaching the objective lens, a beam expander was used to ensure the filling of the back aperture for optimal imaging resolution. The focal spot sizes along the x, y, and z axes were determined using 0.20-μm-diameter fluorescent beads, calculated as the full-width at half-maximum (FWHM) [Fig. 1(b)]. The lateral resolution was measured to be 0.87±0.12  μm, whereas the axial resolution was 4.59±0.48  μm (mean ± s.e.m, n = 5).

### 2.2 Animals

All experimental procedures complied with the project license (PPL number: PP6988384), Home Office, and Imperial College London institutional norms. Heterozygous ${\mathrm{APP}}^{\mathrm{NL}\text{-}G\text{-}F}$ knock-in mice^19^ with one copy of the mutant APP gene were employed as AD models. The humanized Aβ buildup induced by mutations results in similar disease development, making the mouse model suited for studying the link between amyloid plaques and blood vessels. Six mice were used: one female aged 8.9 to 9.9 months and two females aged 6.5 to 7.5 months for chronic imaging, and a 10- and 11-month-old mouse for image analysis pipeline development; a 1-month-old wildtype (C57BL/6) mouse was also imaged for pipeline development.

### 2.3 Cortical Surgery and Viral Injection

Mice were given Carprofen (5  mg/kg) and buprenorphine (0.07  mg/kg) for pre-operative pain management and were operated on under 1.5% to3% isoflurane anesthesia. A heated blanket and a rectal thermometer were used to maintain body temperature at 37°C. A circular craniotomy was made over two fields of view (FOVs) in the cortex. FOV I was located 2.25 mm ML, −3  mm AP from bregma, and FOV II was located 2.25 mm ML, −1 AP mm from bregma [Fig. 1(c)]. The craniotomy was outlined with a 3 mm biopsy punch, and the skull piece was removed using a dental drill. A 3 mm coverslip was attached to a 5 mm coverslip using optical glue and attached onto the craniotomy together with a head plate using dental cement. After surgery, a photograph was taken as a reference for vascular mapping. Mice were allowed to heal for 35 days before beginning imaging.^39^^,^^40^ For one mouse imaged for pipeline development, 5  μL of the virus CAG-NLS-GFP (Addgene 104061, Watertown, Massachusetts, United States, titer $2.5\times 10^{13}\;\mathrm{GC}/\mathrm{ml}$) was injected through the facial vein at P0.^41^ The virus contains a green genetically encoded nuclear fluorescent protein. Imaging was done at P32.

### 2.4 Chronic Imaging

Mice underwent two imaging sessions with a 4-week interval. Mice were anesthetized with 1% to 1.2% isoflurane, maintained at 37°C, and monitored via an infrared camera. Amyloid plaques were visualized using methoxy-X04 (10% DMSO, 45% propylene glycol, 45% saline), injected intraperitoneally 24 h before imaging. Vessels were imaged with Texas-Red (50  mg/kg at week 5, 25  mg/kg at week 9), delivered via tail-vein injection 30 min before *in vivo* imaging. Using a stereomicroscope before imaging, vessel patterns were compared with the vascular images taken immediately after surgery. Vessels exhibiting distinct morphological features such as large diameter, pronounced curvature, bifurcations, or crossings were identified and used as landmarks. Then, the mouse was stabilized under the microscope by fixing it in a holder using the head plate, and these characteristic features were referenced during both imaging sessions to accurately locate the same region using a 10× air-immersion objective (TL 10X-2P, Thorlabs). Once the area was located, it was imaged with a 25× water-immersion objective (XL Plan NA = 1.0, Olympus) [Fig. 1(d)]. During chronic imaging, the power under the objective ranged from 165 to 182 mW, with inter-session power variability for each FOV not exceeding 8%. To assess measurement stability, a neonatal mouse lung slice was imaged at different power levels, and the width of several structures was measured. Size variability was minimal across an 8% power difference and did not exceed 6% (Fig. S1 in the Supplementary Material). Image stacks (512×512  pixels, 500×500  μm FOV, 5  μm step size along the z-axis) were taken with 5 to 7 frames per plane, depending on image quality, using ScanImage.^42^

### 2.5 Image Processing

**Fig. 2:**
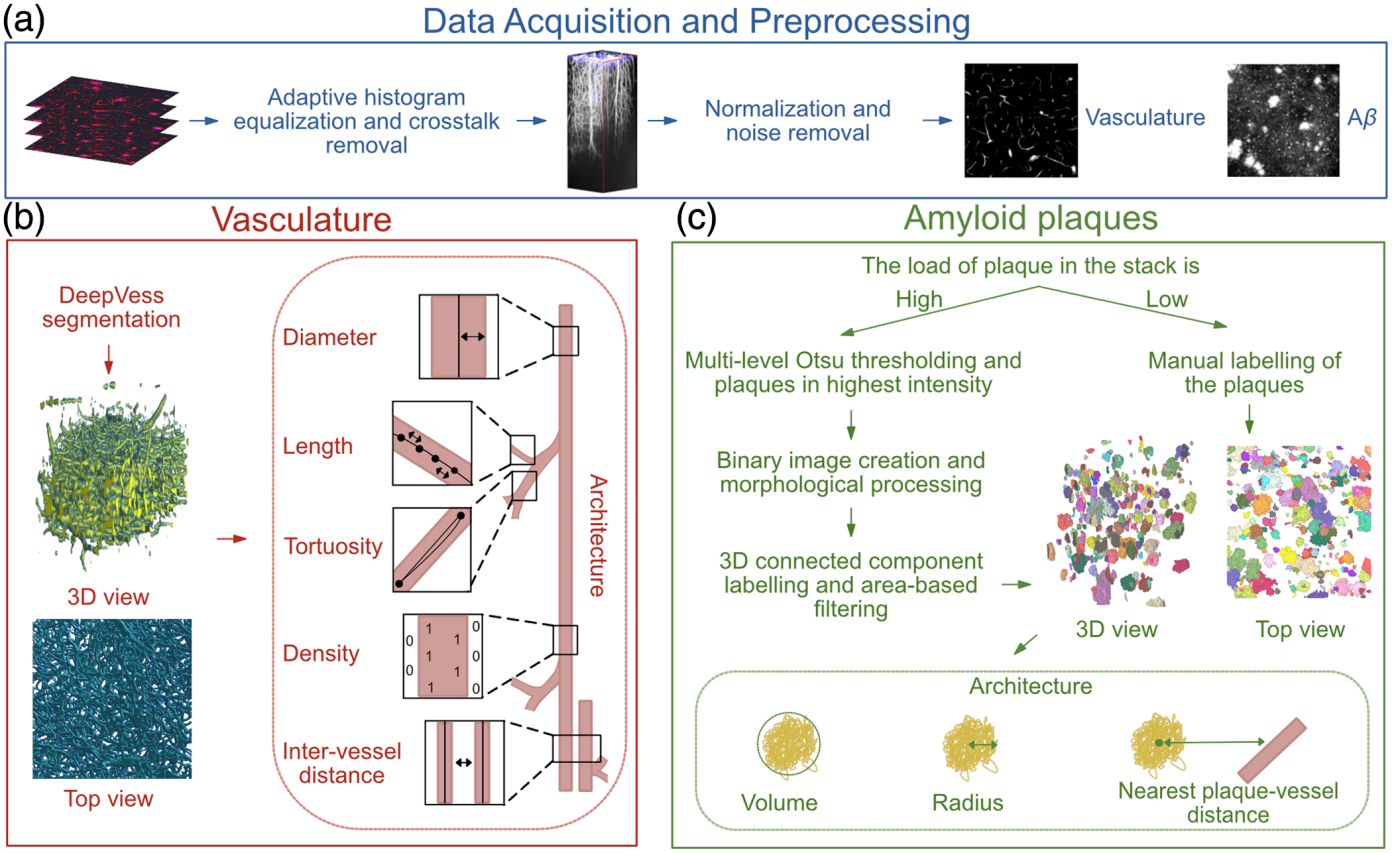
Segmentation and quantification pipeline for blood vessels and amyloid plaques in multiphoton images. (a) Data acquisition and preprocessing: adaptive histogram equalization, crosstalk removal, normalization, and noise. (b) Vasculature analysis: segmentation using the DeepVess pipeline, generating 3D and top-view visualizations. Quantification includes measures of (i) diameter, (ii) length, (iii) tortuosity, (iv) density, and (v) inter-vessel distance. (c) Amyloid plaque analysis: segmentation through multilevel Otsu thresholding for low plaque load or manual labeling for high plaque load. Quantification includes (i) plaque volume, (ii) plaque radius, and (iii) the nearest plaque-vessel distance.

Before processing the images, stacks were manually inspected for obvious signs of signal loss between sessions. Two datasets were excluded from the analysis. Mouse 2, FOV I showed vascular regrowth in session 2, rendering longitudinal comparison with session 1 invalid because of partial signal loss in the FOV. Mouse 3, FOV II exhibited insufficient SNR in both sessions. Images from selected stacks were then intensity-matched using adaptive histogram equalization, and crosstalk was removed. Preprocessing included dura removal, normalization, and noise reduction^25^ [Fig. 2(a)]. Motion artifact was assessed by binarizing the raw data and selecting several single-vascular structures at different depths in each volume, and calculating the shift of the center position of the vascular structure signal brightness of each frame compared with the previous frame (Fig. S2 in the Supplementary Material). The shift was found to be 2 pixels at maximum; therefore, motion correction was not applied. The pipeline was run using MATLAB R2023b and Python.

#### 2.5.1 Blood vessels

Vessels were segmented using the DeepVess 3D convolutional neural network (CNN) model,^25^ which consists of six 3D convolutional layers with ReLU activation and max-pooling, followed by a softmax layer for classification. Dice similarity coefficients were computed for each stack by manually annotating six slices spaced 100  μm apart to provide representative validation across cortical depth. When the Dice coefficient was below 0.7, the corresponding stack was manually corrected using Napari^43^ to improve segmentation accuracy. The Dice coefficients are reported in Table 1.

**Table 1:** Dice similarity coefficients (DeepVess versus manual) by mouse, field of view (FOV), and session. A QC threshold of 0.70 was used to flag cases for manual correction.

Mouse	FOV	Session	Dice	QC action
Mouse 1	I	Session 1	0.83	Accepted
Mouse 1	I	Session 2	0.70	Accepted
Mouse 1	II	Session 1	0.80	Accepted
Mouse 1	II	Session 2	0.74	Accepted
Mouse 2	II	Session 1	0.79	Accepted
Mouse 2	II	Session 2	0.72	Accepted
Mouse 3	I	Session 1	0.53	Manual correction
Mouse 3	I	Session 2	0.63	Manual correction

After segmentation, vessel parameters, including diameter, length, tortuosity, inter-vessel distance, and density, were calculated based on previous works^44^^–^^47^ [Fig. 2(b)]. The diameter D of a vessel segment was determined as twice the median radius measured along the vessel’s centerline, where the radius $r_{j}$ at each point was the minimum distance to the nearest background voxel:

$$r_{j}=\min(\mathrm{dist}(v_{j},\text{background})).$$ (1)

Here, $v_{j}$ is the voxel at the j’th point along the vessel. The diameter D is then given by

$$D=2\times \text{median}(r_{j}).$$ (2)

The length L of a vessel segment was calculated by summing the Euclidean distances between consecutive points along the vessel centerline:

$$L=\overset{N-1}{\underset{i=1}{\sum }}\sqrt{(x_{i+1}-x_{i}{)}^{2}+(y_{i+1}-y_{i}{)}^{2}+(z_{i+1}-z_{i}{)}^{2}},$$ (3)

where $\left(x_{i},y_{i},z_{i}\right)$ are the coordinates of the i’th point along the vessel, and N is the total number of points. Tortuosity T was defined as the ratio of the vessel segment’s path length L to the straight-line distance between its endpoints

$$T=\frac{L}{\sqrt{(x_{N}-x_{1}{)}^{2}+(y_{N}-y_{1}{)}^{2}+(z_{N}-z_{1}{)}^{2}}}.$$ (4)

Here, $\left(x_{1},y_{1},z_{1}\right)$ and $\left(x_{N},y_{N},z_{N}\right)$ represent the coordinates of the starting and ending points of the vessel segment, respectively. The total vascular volume ($V_{\text{vessel}}$) was computed as the number of annotated (nonzero) voxels $N_{\text{vessel}}$ multiplied by the physical volume represented by a single voxel $V_{\text{voxel}}$

$$V_{\text{vessel}}=N_{\text{vessel}}\times V_{\text{voxel}}.$$ (5)

This physical volume $V_{\text{voxel}}$ is given by the product of the in-plane pixel size and axial spacing:

$$V_{\text{voxel}}=\Delta x\times \Delta y\times \Delta z.$$ (6)

Finally, the inter-vessel distance (IVD) was calculated using a single midpoint per vessel segment. Specifically, for each skeletonized segment, we took the centroid (mean of its [x,y,z] skeleton coordinates). We then measured Euclidean distances from that centroid to the centroids of all other segments and reported the minimum of these distances for that segment:

$${\mathrm{IVD}}_{ij}=\underset{v}{\min}\sqrt{(x_{ij}-x_{v}{)}^{2}+(y_{ij}-y_{v}{)}^{2}+(z_{ij}-z_{v}{)}^{2}}$$ (7)

where $\left(x_{ij},y_{ij},z_{ij}\right)$ are the coordinates of the midpoint of vessel segment ij, and $\left(x_{v},y_{v},z_{v}\right)$ are the coordinates of the nearest segment midpoint v.

### 2.6 Amyloid Plaques

A Python script was developed to segment Amyloid plaques either semi-automatically using the SciPy package^48^ with manual correction via Napari,^43^ or fully manually [Fig. 2(c)]. Semi-automatic segmentation was used when the data showed more than 45 plaques with good imaging quality; otherwise, manual annotation was performed. For semi-automatic segmentation, four intensity thresholds were generated using multilevel Otsu thresholding, with plaques identified as regions with the two highest intensities. A binary image was then created, and morphological operations filled small holes and removed small objects. Area-based filtering was applied after 3D-connected component labeling. Regions smaller than $49\;\mu m^{2}$ were excluded.^49^ The 500×500 labeled areas in 2D planes (XY) with a 0.98  μm pixel size were summed and transformed into 3D using a 5  μm (Z) step size. The volume of each plaque was calculated directly from the 2D segmentations by summing per-slice areas and multiplying by the slice thickness. Using that volume, a radius was computed as the equivalent-sphere radius from this measured volume for comparison purposes.

### 2.7 Nearest Plaque-to-Vessel Distance

The coordinates of plaque centroids were retrieved from the segmented 3D binary data set. The closest neighbouring vessel of each centroid was identified using 3D Euclidean distances analysis.^50^

### 2.8 Data Analysis

Python was used for statistical analysis using the SciPy^48^ and Scikit-learn^51^ packages. The signal-to-background ratio (SBR) of imaging stacks was determined as the average of the brightest 15 and darkest 15 pixels in each profile.^52^ The signal strength was quantified by averaging the intensity of the top 0.5% of pixels in each frame.^32^ Data were tested for normality before statistical testing of vascular parameters and amyloid plaques between imaging sessions within a mouse. Mann–Whitney U tests were used as statistical testing. We reported distribution means as sample mean ± standard error of the mean unless specified otherwise.

## 3 Results

### 3.1 3PM Imaging: Dual-Channel Visualization of Vessels with Amyloid Plaques or Neurons

**Fig. 3:**
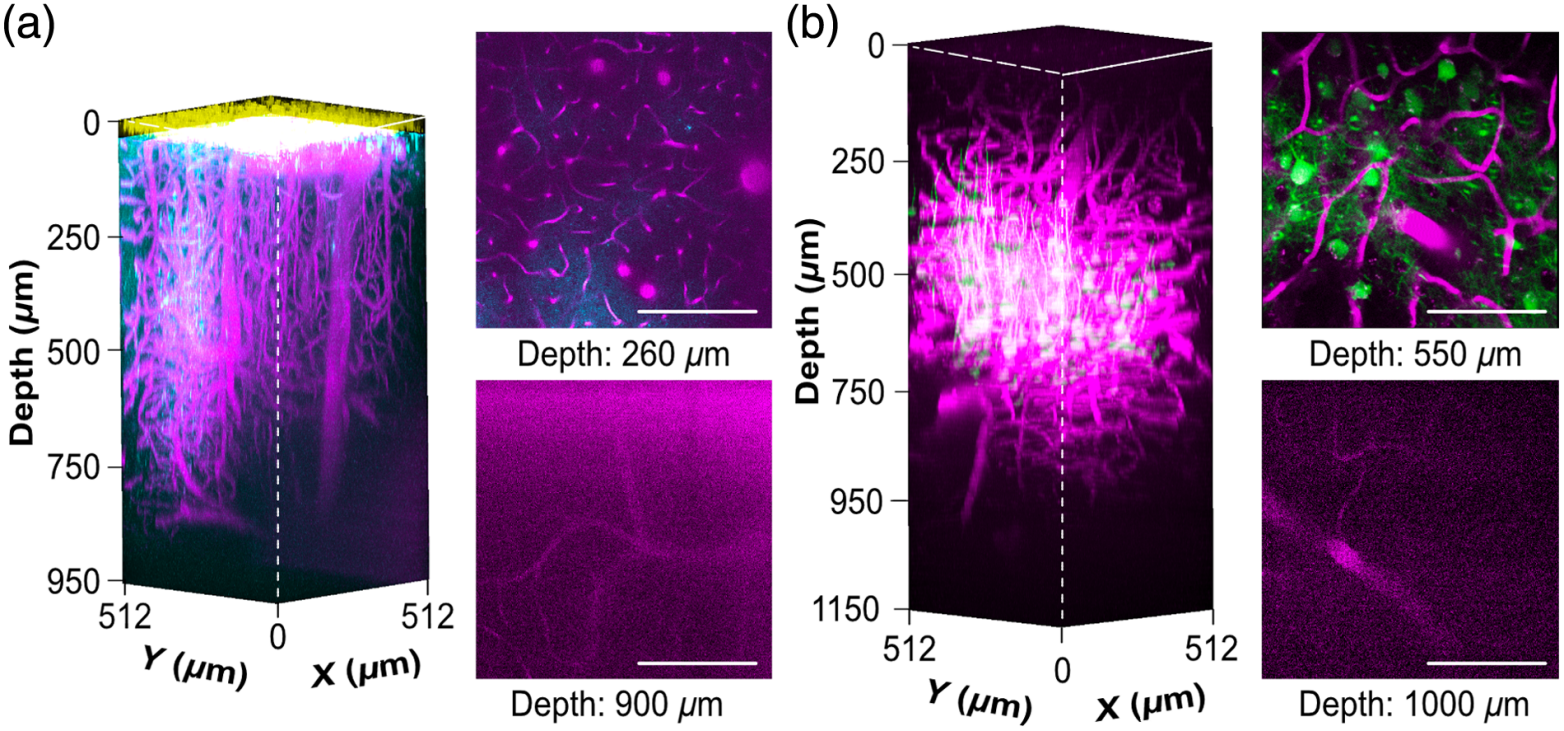
Multicolor 3P fluorescence images using 1340-nm excitation. 3D reconstruction of (a) vessels dyed with Texas Red (magenta), plaques with methoxy-XO4 (cyan), and THG (yellow) image stacks taken with 5  μm z-steps, and (b) vessels dyed with Texas Red (magenta) and neurons labeled with GFP (green) taken with 10  μm z-steps. Stacks are normalized by adjusting the intensity of each slice based on the intensity range of the last nonoverexposed slice (95% of maximum brightness) to ensure consistent brightness across the stack. The maximum laser power was (a) 182 mW and (b) 200 mW under the objective lens. Scale bars: 200  μm.

Using 1340-nm three-photon (3P) excitation, we acquired deep-tissue fluorescence images of the mouse cortex labeled with multiple fluorescent probes [Fig. 3]. In one configuration, vessels were visualized by Texas Red (magenta), whereas amyloid plaques were selectively stained with methoxy-XO4 (cyan). An additional signal from third-harmonic generation (THG, shown in yellow) shows the dura mater. In a second configuration, Texas Red (magenta) was used to label the vasculature, whereas neurons labeled with GFP (green) highlighted neuronal structure. The resulting 3D renderings captured both vascular architecture and either plaque or neuronal features in a single scan, underscoring the utility of 3PM for simultaneous, deep-tissue imaging of multiple tissue components.

### 3.2 Consistent Field of View Tracking Across Two Imaging Sessions

**Fig. 4:**
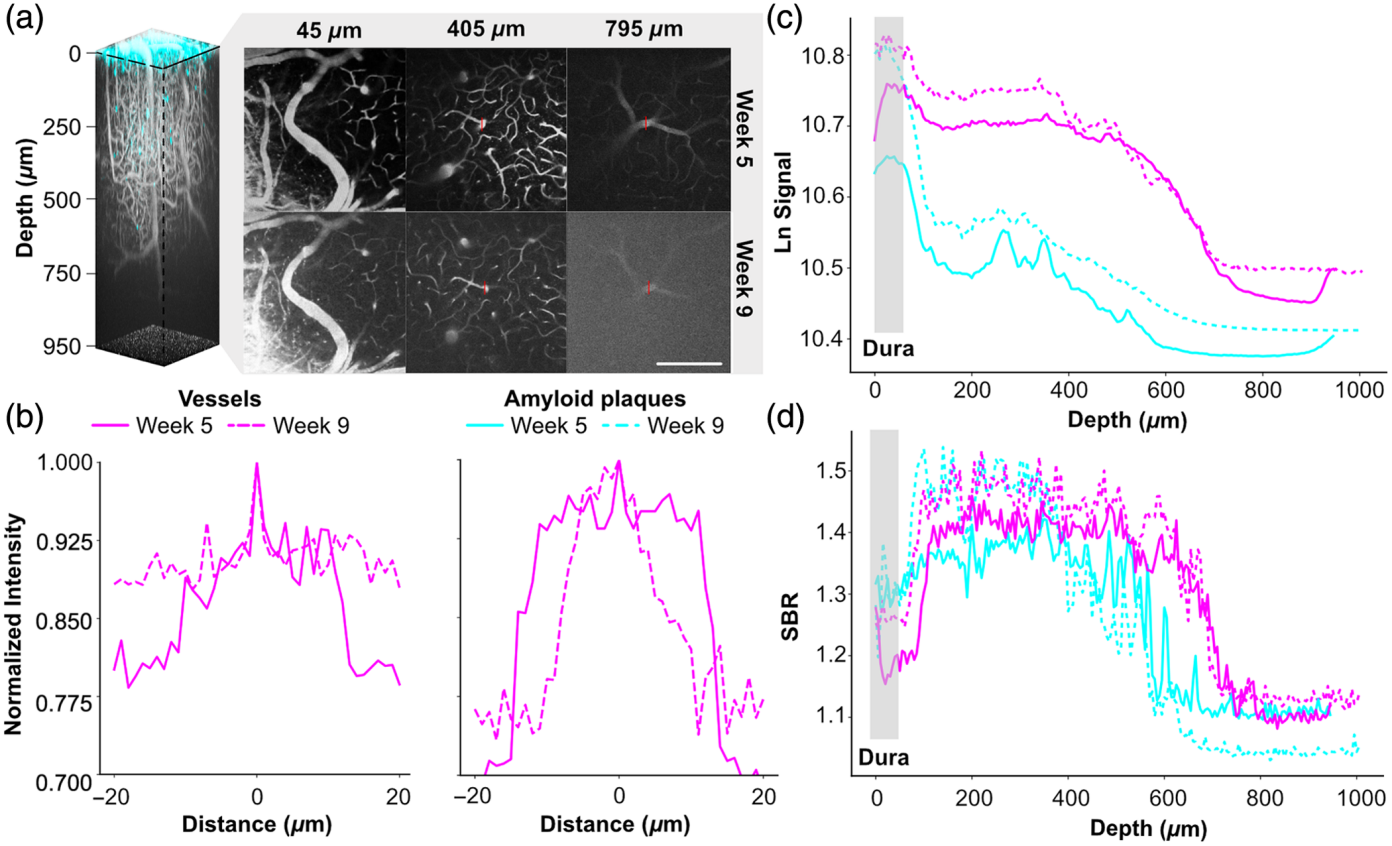
Signal-to-background ratio (SBR) and signal strength analysis over depth. (a) 3D reconstruction of vessels and plaques image stack. The amyloid plaques are labelled with methoxy-XO4 (blue), and the vessels are labelled with Texas Red (grey). The excitation wavelength is 1340 nm, the maximum laser power was 182 mW under the objective lens, and images were taken with 5  μm z-steps. Shown are images taken with a 25× objective lens at different depths (45, 405, and 795  μm). The brightest vessel at 405 and 795  μm is marked in red. (b) Line profiles of the vessels highlighted with the brightest markers in red at depths 405 and 795  μm. Scale bars are equal to 200  μm. (c) The signal strength of the vessels was quantified by averaging the intensity of the top 0.5% of pixels in each frame.^32^ (d) The SBR was determined as the average of the brightest 15 and darkest 15 pixels in each profile. The SBR decreased with increasing depth, and there was an overall higher SBR in the first imaging session.^52^

We conducted longitudinal imaging of the same FOV in ${\mathrm{APP}}^{\mathrm{NL}\text{-}G\text{-}F}$ knock-in mice at weeks 5 and 9 after surgery to assess the progression of vascular and amyloid pathologies [Fig. 4(a)]. Using vascular landmarks identified in postoperative bright-field images, we localized the FOV with a 10× air-immersion objective. High-resolution imaging was performed with a 25× water-immersion objective, enabling 3PM of cortical depths ranging from 45 to 795  μm.

To evaluate the reproducibility and depth-resolved imaging capabilities of our 3PM setup, we analyzed intensity profiles of the brightest vessels at depths of 405 and 795  μm across both imaging sessions [Fig. 4(b)]. The brightest vessels were identified by averaging the intensity of the top 0.5% of pixels in each image slice. Normalized line profiles demonstrated consistent peak intensities at the same spatial locations between sessions, confirming reliable re-identification of vascular structures over time.

We quantified signal intensity and SBR for both vascular and amyloid signals as a function of imaging depth [Figs. 4(c) and 4(d)]. Vessel signals exhibited consistently higher SBR values compared with amyloid plaques at all depths, indicating superior contrast and detectability for vascular structures during deep imaging. Both signals displayed an initial increase near the cortical surface, attributed to higher tissue density in the dura mater, followed by a decline with depth due to light scattering and absorption. The dura mater location was determined using the THG signal at the top of the stack. Variations in signal strength and SBR between sessions were primarily attributable to differences in laser power settings and dye concentrations (Texas Red for vessels and methoxy-XO4 for amyloid plaques). To minimize these effects, raw data were processed using adaptive histogram normalization, and the dura mater was excluded during segmentation to avoid artifacts.

### 3.3 Overall Vessel Volume Decreased and Amyloid Plaques Increased in the Second Session

**Fig. 5:**
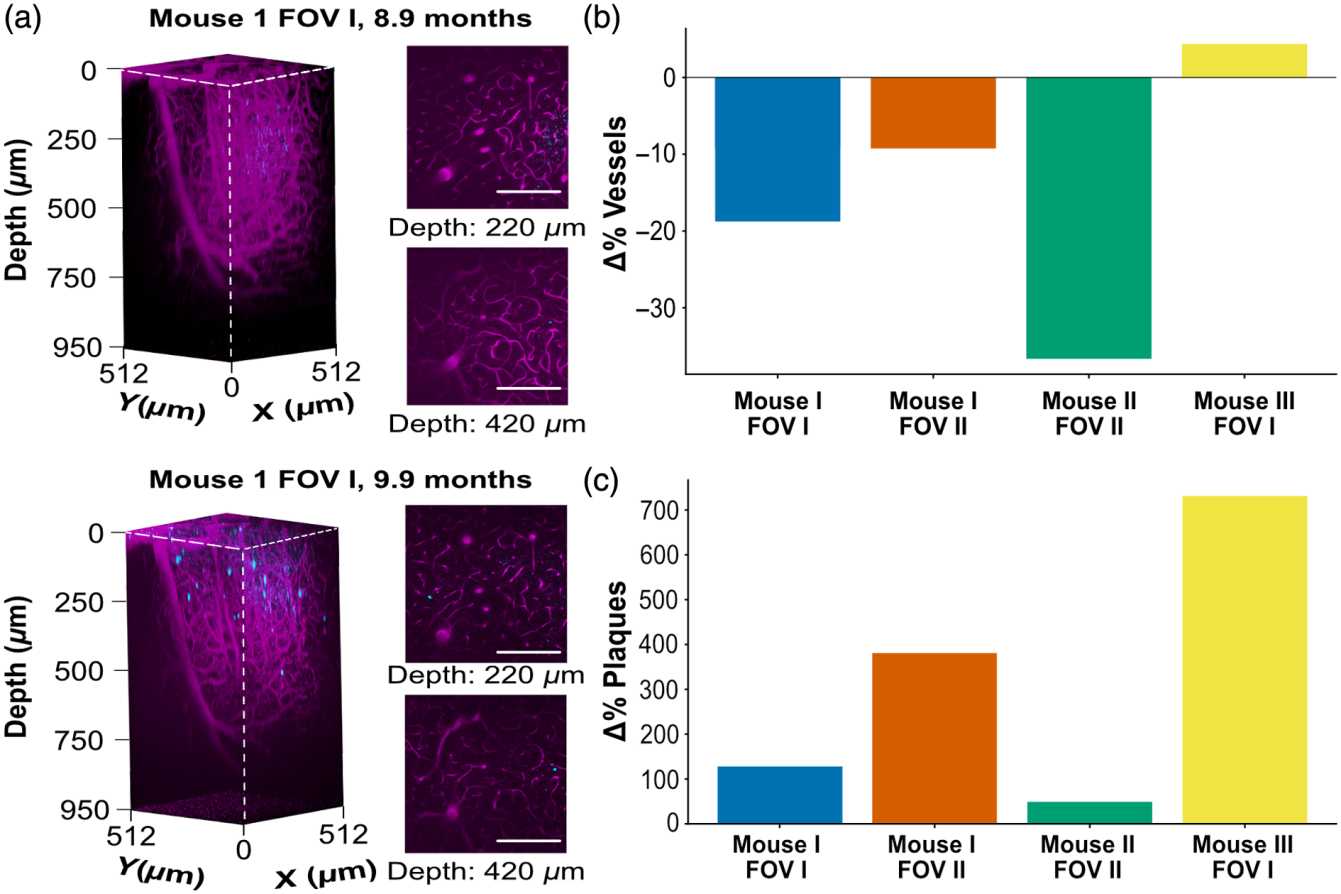
Vessel and amyloid plaques volume changes between imaging sessions 1 and 2. (a) Representative three-photon fluorescence renderings of vasculature (magenta) and amyloid plaques (cyan) from mouse 1 FOV I at session 1 (top) and session 2 (bottom), with example slices shown at 220 and 420  μm depth. (b) Percent change in vessel volume (S2 versus S1) across four longitudinally matched FOVs from three mice. (c) Percent change in plaque volume (S2 versus S1). Each bar represents one FOV. Scale bars are 200  μm.

After segmentation, vascular and amyloid volumes were quantified across two imaging sessions in four longitudinally matched FOVs from three mice aged 6.5 to 9.9 months [Fig. 5]. Percent change in vessel volume between sessions (S2 vs. S1) ranged from −36.6% to 4.3%, whereas percent change in plaque volume ranged from 48.2% to 730.2%. The data show a vessel volume decrease in 3 FOVs, whereas the plaque volume increased in all FOVs.

### 3.4 Significant Vessel and Plaque Changes across Sessions

**Fig. 6:**
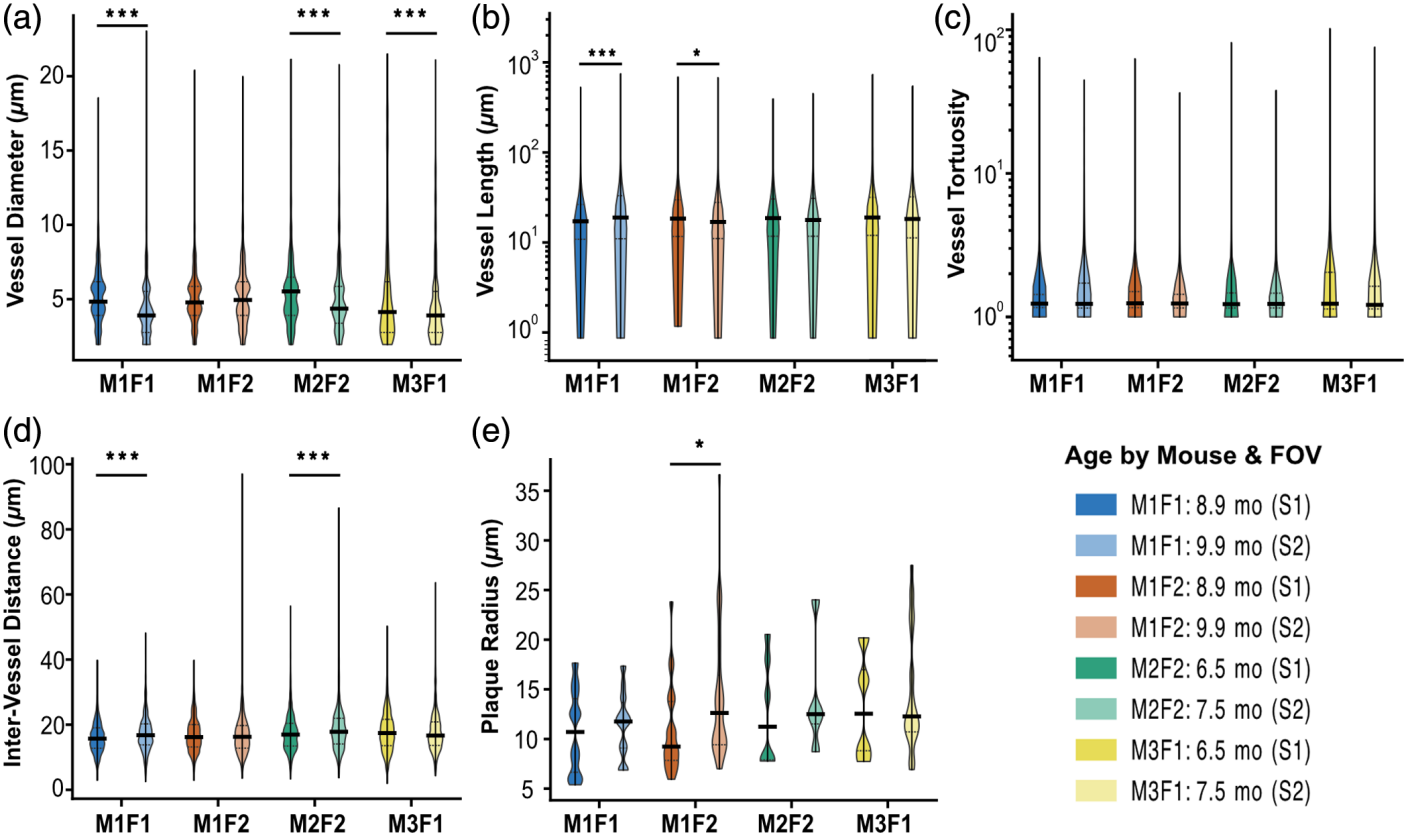
Change of vascular parameters between imaging sessions 1 and 2. Subplots show (a) vessel diameter, (b) vessel length, (c) vessel tortuosity, (d) inter-vessel distance, and (e) plaque radius. Data were tested for normalization, and statistical analysis was done using the Mann–Whitney U test. p values are indicated by stars, *p<0.05, **p<0.01, and ***p<0.001.

As shown in Fig. 6, vessel diameter decreased significantly between the two imaging sessions for three of the four FOVs (Mann–Whitney U tests: all p<0.001). Vessel length significantly increased for mouse 1 FOV 1 (p<0.001) and decreased FOV 2 (p=0.031), whereas no significant difference was observed for mouse 2 FOV 2 or mouse 3 FOV 1 (p>0.05). Vessel tortuosity did not differ significantly between sessions for any FOV (all p>0.05). Inter-vessel distance increased in the second session for both mouse 1 FOV 1 and mouse 2 FOV 2 (both p<0.001). Plaque radius significanlty increased only for mouse 1 FOV 2 (p<0.05), with no other significant differences observed across FOVs (p>0.05).

## 4 Discussion

In this proof-of-concept study, we established a longitudinal three-photon microscopy (3PM) approach for imaging vascular and amyloid pathologies in deep cortical structures of ${\mathrm{APP}}^{\mathrm{NL}\text{-}G\text{-}F}$ knock-in mice, a model of AD. This methodology overcomes the depth limitations of two-photon microscopy (2PM) and enables *in vivo* imaging of cortical structures at high resolution over extended periods. Although the present dataset is limited in size (four FOVs in three animals) and duration, it demonstrates the feasibility of using 3PM to monitor structural changes associated with AD pathology over time with increased sample size.

### 4.1 Longitudinal Three-Photon Imaging of Cortical Structures

Our imaging methodology enabled longitudinal visualization of amyloid plaques at cortical depths of up to 420  μm, together with vascular structures observable to depths of up to 920  μm [Fig. 3(a)], which exceeds the previously reported range of 150 to 200  μm achieved in *in vivo* studies of amyloid plaques using two-photon microscopy (2PM).^30^^,^^37^ Although longitudinal studies using MRI and PET have achieved greater imaging depths,^53^^,^^54^ they do so at a significantly lower resolution. 3PM, with its cellular resolution, bridges the gap between superficial multiphoton imaging and macroscopic modalities such as MRI and PET and has the capability to analyze neuronal circuit activity in addition to amyloid plaque load and vasculature.

In this paper, we achieved a maximum imaging depth of 1.09 mm [Fig. 3(b)] in a 5-week-old mouse, which is consistent with the previously reported depth of 1.1 mm by Ouzounov et al.,^31^ using the same excitation wavelength of 1340 nm. In older animals (6.5 to 9.9 months), the attainable imaging depth was reduced, likely caused by increased light scattering in more mature brain tissue, limiting optical penetration.^55^. Improvements can possibly be expected with the use of longer wavelengths or optimized immersion media such as deuterium oxide ($D_{2}O$).^36^ Imaging of AAV-mediated GFP expression alongside vascular labeling confirmed spectral compatibility and minimal cross-talk at 1340 nm, supporting the feasibility of multicolor 3PM for simultaneous imaging of genetically and chemically labeled structures.

Field-of-view consistency across imaging sessions was ensured using vascular landmarks and stable mounting techniques [Fig. 4(a)], with normalized line profiles demonstrating reproducibility [Fig. 4(b)]. This highlights the feasibility of 3PM for longitudinal studies, offering high resolution with minimal photodamage due to longer excitation wavelengths and low average laser power.^33^ The combined use of Texas Red–dextran and methoxy-X04 for simultaneous in vivo visualization of cerebral vasculature and amyloid plaques is well established and has been validated in prior multiphoton imaging studies.^56^ However, future studies aiming to further integrate structural and molecular readouts could incorporate post-imaging immunohistochemical staining. CD31 for endothelial cells and Thioflavin-S or 6E10 for amyloid plaques could be used to compare longitudinal *in vivo* dynamics with fixed-tissue histopathology, thereby deepening the biological interpretation of three-photon datasets.^57^^,^^58^

### 4.2 Vascular Degeneration and Amyloid Accumulation

Although biological interpretations cannot be drawn from this limited dataset, the longitudinal imaging pipeline captured significant variations in vascular and amyloid volumes over time (Figs. 5 and 6). Prior studies in AD models have consistently reported reductions in vessel density and diameter, increased inter-vessel spacing, and general vascular rarefaction accompanying amyloid accumulation.^59^^–^^70^

It should be noted that apparent vascular changes could be influenced by technical or physiological factors such as minor compression during imaging, tissue swelling, or variability in vascular dye perfusion. Furthermore, the 4-week imaging interval may be insufficient to capture slow vascular remodeling relative to amyloid deposition. The relatively subtle plaque-associated effects are consistent with the slower progression characteristic of the heterozygous NL-G-F genotype compared with more aggressive or homozygous AD models.^22^^,^^23^

Overall, this pilot dataset demonstrates that 3PM can quantify vascular and amyloid structures in the deep cortex across imaging sessions, establishing a foundation for future studies. Expanding this approach to larger sample sizes, multiple cortical regions, and longer monitoring intervals will allow quantitative assessment of the temporal relationship between vascular remodeling and amyloid accumulation in AD and possibly its response to therapeutic interventions.

## 5 Conclusion

We developed a longitudinal 3PM imaging methodology for studying vascular and amyloid pathologies in deep cortical structures of ${\mathrm{APP}}^{\mathrm{NL}\text{-}G\text{-}F}$ mice. This approach could provide detailed insights into AD progression and surpasses traditional depth limitations of 2PM. Future research should leverage this methodology with larger cohorts, extended monitoring periods, and advanced analytical tools, such as machine learning algorithms, to further elucidate the interplay between vascular and amyloid pathologies in AD and evaluate potential therapeutic strategies.

## Supplementary Material

10.1117/1.JBO.31.1.016004.s01

## Data Availability

Code used to generate the figures in this paper can be found on https://github.com/estassss/Longitudinal-three-photon-imaging. Data are available on https://doi.org/10.5061/dryad.wh70rxx2j.
