# Supplementary material for: Longitudinal three-photon imaging for tracking amyloid plaques and vascular degeneration in a mouse model of Alzheimer’s disease
Source: J Biomed Opt. 2026 Jan 2;31(1):016004. doi: 10.1117/1.JBO.31.1.016004 (PMC12771024; doi:10.1117/1.JBO.31.1.016004)
Supplement: Supplementary file 1 [file JBO_031_016004_SD001.pdf]

Supplementary Material

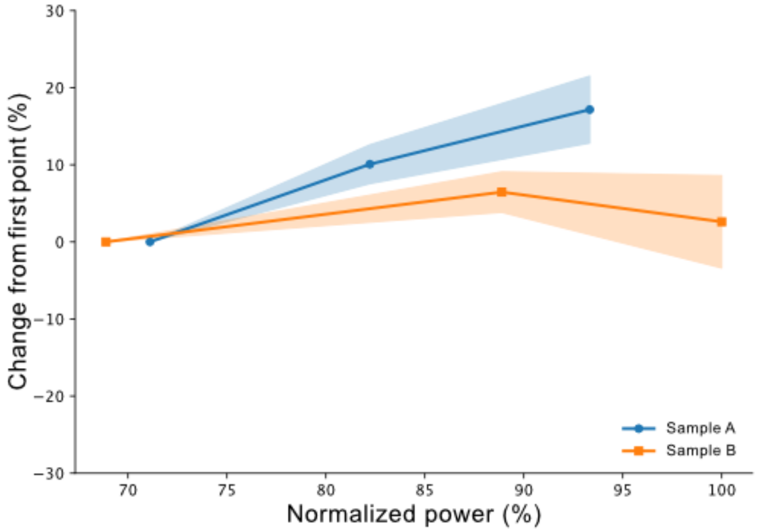

Fig S1. Stability of measurement. Measured width of structures in a neonatal mouse lung slice imaged under varying laser power levels.

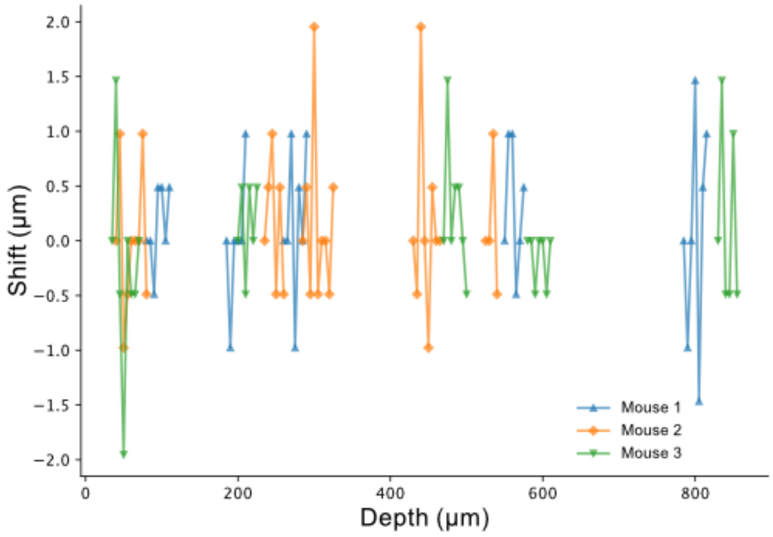

Fig S2. Motion artifact assessment. Shift of selected vessels (relative to previous slice) per depth of FOV 1 Session 1 for the three mice.
